# Aerosolized Insecticide Spray Distributions and Relationships to Efficacy against Stored Product Pests

**DOI:** 10.3390/insects14120914

**Published:** 2023-11-28

**Authors:** Daniel Brabec, Srinivas Lanka, James F. Campbell, Frank H. Arthur, Deanna S. Scheff, Kun Yan-Zhu

**Affiliations:** 1 Center for Grain and Animal Health Research, Agricultural Research Service, USDA, 1515 College Ave., Manhattan, KS 66502, USAdeanna.scheff@usda.gov (D.S.S.); 2 Department of Entomology, Kansas State University, Manhattan, KS 66502, USA

**Keywords:** hedging, transaction costs, dynamic programming, risk management, post-decision state variable

## Abstract

**Simple Summary:**

Aerosol insecticides are widely used in food processing and warehouse facilities to help control insect contamination of grain and packaged products. These tests were conducted in the experimental flour mill at a regional university. One insecticide, Turbocide^®^, was applied from a high-pressure cylinder. The nozzle pressure was ~800 psi, and the spray plumes were very fine (VMD ~18 µm). The other insecticide, Pyrocide^®^, was applied using a hand-held fogging system, which delivered a larger distribution of droplets (VMD ~50 µm). These insecticides were dispensed from varied locations on the milling floor. The spray applications were monitored with droplet counting instruments and bioassay dishes with either adult or larvae flour beetles. Droplet data were summarized into two measurements: mass concentration index (MCI) and deposition index (Dep.Idx). Contour plots of Dep.Idx values were developed and were useful to show and compute the amount of floor area that was poorly treated. For Turbocide, adult insect efficacy increased with increasing MCI and Dep.Idx values, but larval insect efficacy was high at all MCI and Dep.Idx values. In contrast, with Pyrocide, there was no apparent relationship between aerosol MCI or Dep.Idx and adult insect efficacy. However, with Pyrocide, there was a positive relationship between the larval efficacy and the Dep.Idx. These results are useful in developing more effective application strategies for optimum insect control.

**Abstract:**

Aerosol insecticides are widely used in stored product insect management programs in food facilities. Previous research has shown spatial variation in aerosol efficacy within facilities, but information on how spatial patterns of aerosol droplet concentration, size distribution, dispersal, and deposition contribute to this variation in efficacy is limited. This study involved two aerosol application systems: a high-pressure cylinder containing TurboCide Py-75^®^ with pyriproxyfen IGR (ChemTech Ltd., Des Moines, IA, USA) and a hand-held fogger containing Pyrocide 100^®^ (MGK, Minneapolis, MN, USA) with Diacon II which contains methoprene IGR (Wellmark, Schaumburg, IL, USA). These systems were used at single or multiple application locations. The spray trials were conducted in a small-scale flour mill, Hall Ross Flour Mill (Kansas State University, Manhattan, KS, USA). The droplet size distributions were monitored at multiple positions within the room using nine aerodynamic particle sizing (APS, TSI Incorp, Shoreview, MN, USA) instruments. The APS data collected over the treatment period were summarized into a mass concentration index (MCI), which ranged from 155 to 2549 mg/m^3^ for Turbocide and 235–5658 mg/m^3^ for Pyrocide. A second parameter called the Deposition Index (Dep.Idx) was derived to estimate potential insecticide depositions on the floor and has units of g/m^2^. The Dep.Idx was below 5.3 g/m^2^ for most Turbocide applications, while the Dep.Idx was below 8.4 g/m^2^ for most Pyrocide applications. The MCI and Dep.Idx values varied with APS position and spray application location, with proximity to the aerosol application location and degree of obstruction between the release point and APS position contributing to this variation. We assessed the relationship between aerosol droplet parameters and insect efficacy using *Tribolium confusum* Jacqueline DuVal, the confused flour beetle. The adults were treated directly, while the larvae were treated two weeks later during the residual test (previously published). For Turbocide, efficacy against adults increased with MCI and Dep.Idx values, but for residual efficacy of the IGR, efficacy was high at all aerosol droplet values, so no relationship was apparent. In contrast, the relationship between Pyrocide deposition and adult insect efficacy was highly variable. But with larval insect efficacy, residual larvae control was directly related to increases in Pyrocide MCI and Dep.Idx. Contour plots of Dep.Idx values were developed, which could be used to predict areas of the mill that are not receiving an adequate application rate, and this could be used to develop more effective application strategies for aerosol insecticides in food facilities.

## 1. Introduction

The use of aerosolized insecticides as a management tool for stored product insects in food facilities has a long history. However, aerosol usage has increased in recent years due in part to decreased use of structural fumigation resulting from the phase-out of the fumigant methyl bromide [1,2,3,4]. Aerosol insecticides do not replace fumigants since they have low penetration ability but can be an important component of integrated management programs (IPM) for mills and other food processing and storage facilities [5,6,7]. Aerosol applications can be an alternative to conventional insecticide spot or surface treatments because they are better able to disperse and settle on the surfaces in the three-dimensionally complex landscapes found in food facilities. This improved coverage increases the likelihood of stored product insects encountering the insecticide either during application or through contact with residual insecticide deposition on surfaces.

There are a range of aerosol insecticide formulations and application methods for applying aerosol insecticides. Application methods vary based on the mechanism for producing the force to move the liquid insecticide through atomizing nozzles to produce the small-sized droplets. Application systems can utilize pressurized cylinders with carrier gases attached to spray nozzles, compressed air routed through permanently installed overhead spray nozzles, or portable or hand-held sprayers with pumps to force liquid through a nozzle. Different aerosol application methods and insecticide formulations produce different droplet size distributions and concentrations, as well as different release velocities. Droplet size distribution and concentration at a specific location within a facility are impacted by not only the application method but also by the distance from the application release point, the structural configuration of the food facility, and environmental conditions [5,6,8,9]. A study in a pilot-scale flour mill, (Hall Ross Flour Mill Kansas State University, Manhattan, KS, USA), demonstrated that when an insecticide was dispensed from a pressurized cylinder with pyrethrin and an insect growth regulator, pyriproxyfen (ChemTech Ltd., Des Moines, IA, USA), the average geometric mean diameter (GMD) of settled droplets decreased with the distance from the application point [8]. 

In aerosol insecticide applications, aerosol droplet size is a critical factor influencing droplet deposition and efficacy. Larger droplets settle out of the air more rapidly than smaller droplets, while very small droplets tend to remain suspended in the air for long periods of time [10]. Thus, they are less likely to impinge on surfaces and contribute to insecticide efficacy. In a vertical air flow chamber designed to produce a controlled volumetric median diameter (VMD), 16 µm droplets were found to be more efficacious against adult *Tribolium confusum* Jacqueline DuVal, the confused flour beetle, than 2 µm droplets when insects were directly exposed to the insecticide, and additional studies have shown similar trends using a range of droplet sizes and insect species [11,12,13]. Similar tests with 2 and 16 µm sprays were performed on psocid nymphs, which demonstrated improved efficacy with the larger spray droplets [14]. Vertical flow chambers, (MRIGlobal, Kansas City, MO, USA), rely on a continuous flow of consistent droplet size distribution and concentration, but applications in the field typically release droplets over a short period of time, with concentration and size distributions changing with time and with distance. 

Spatial variation in aerosol insecticide efficacy on stored product insects was previously observed in food facilities, which was likely due to spatial variation in aerosol droplet size distribution and concentration impacting deposition [9]. However, there was limited information available on droplet characteristics (size distribution and concentration) during actual aerosol treatments in spatially complex food facilities and on the relationships between droplet characteristics and insect efficacy. Arthur et al. [8] evaluated aerosol droplet distribution at three locations along a transect on a different floor of the same mill used in this study, and Scheff et al. [3] measured aerosol insecticide droplet size distribution and concentration, as well as direct and residual efficacy, inside a rice mill during a single aerosol treatment. The objectives of the current experiment were to (1) assess aerosol droplet characteristics at different locations within a mill during aerosol applications; (2) evaluate how aerosol insecticide application systems, formulations, and application locations impact the spatial distribution of droplets; (3) assess different methods to quantify and analyze droplet count and size data in relation to the potential to settle onto surfaces; and (4) determine relationships between aerosol droplet data and insect efficacy data (both direct mortality and residual efficacy). The insect control efficacy data were previously reported [6,15], but they are presented again here to assess the relationship between efficacy and aerosol droplet data. 

## 2. Materials and Methods

**Figure 1:**
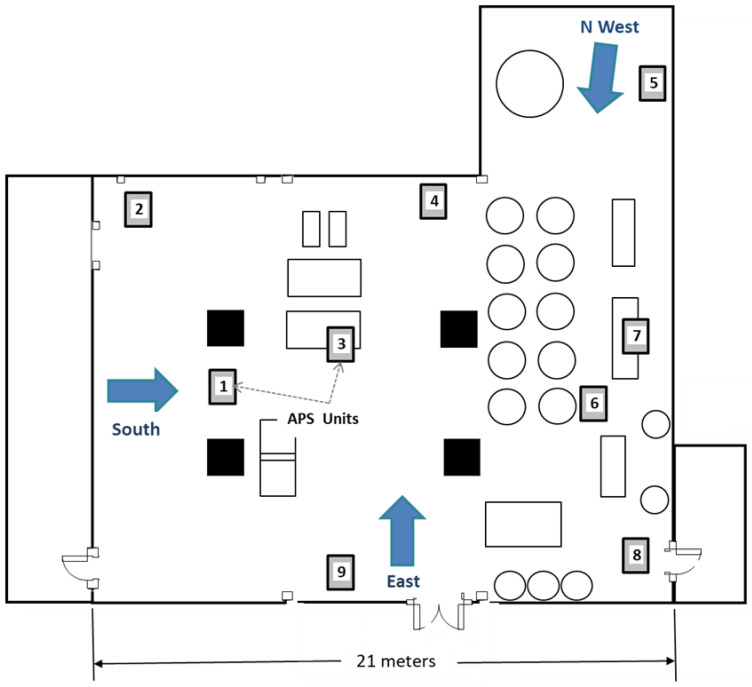
Schematic diagram of the third floor of the pilot-scale flour mill with the positions of the nine Aerodynamic Particle Sizer (APS) instruments (gray squares with numbers 1–9), the three pesticide spray locations (gray arrows and labeled 1-South, 2-East, and 3-NWest), equipment and bins (rectangular and circular symbols), and structural pillars (black squares).

Experiments were carried out at the pilot-scale flour mill at Kansas State University (Hal Ross Flour Mill, KSU), Manhattan, KS, USA. The mill is a concrete building comprising five floors (described in more detail in Campbell et al. [9]). In this study, only the third floor was used. This floor is L-shaped, with the main area measuring ~13.5 by 21 m and the side area in the northwest corner measuring 7.5 by 6.5 m. The ceiling height was 4.3 m, and the total room volume was ~1500 m^3^ (Figure 1). The floor contained structural pillars and multiple pieces of equipment, ducting and piping, and storage bins. Doorways, ducts, and miscellaneous openings between the floors were sealed off using plastic sheeting during the treatments. The temperature during the testing period ranged between 27 and 30 °C with ~60% relative humidity. 

### 2.1. Insecticides and Application Systems

Two aerosol insecticide formulations and application systems were used in this study, which are consistent with those used in previous research evaluating aerosol efficacy at this facility [1,5,9,15]. The formulations and methods of application chosen represent the two commercial options for conducting an aerosol application. The formulations have some similarities in that both contain pyrethrin and both contain the synergist piperonyl butoxide. The methods of application rely on the insecticide manufacturer and the insecticide label. The two methods of application, cylinderized vs. mechanical fogger, are the only two methods currently available to produce particles in the range of 5–50 µm, which has been defined as the particle size range for aerosols [10]. Cylinderized applications are generally for large spaces within long warehouses and processing facilities, while mechanical foggers are for smaller/tighter spaces and focused applications.

The first aerosol evaluated was TurboCide Py-75^®^ with IGR (ChemTech Ltd., Des Moines, IA, USA). This is a combination of pyrethrins (0.7% pyrethrins (AI), 5.0% piperonyl butoxide (synergist), 0.27% pyriproxyfen (AI), and 94% other ingredients) and the insect growth regulator (IGR), pyriproxyfen, trade name NyGuard. Hereafter, this aerosol insecticide will be referred to as Turbocide. The application system for Turbocide was a high-pressure gas cylinder containing CO_2_ as a propellant for dispensing the insecticide. This dissemination system had a pair of cone nozzles plumbed to the outlet of the cylinder (Spraying Systems, Glendale Heights, IL, USA, model 1/8 MEG-0002, ~0.9 mm dia. orifice). The cylinder was set on the floor with the nozzle at a height of ~0.7 m. The nozzles of the cylinder were directed upward at ~30° during application and slowly moved left to right while dispensing the insecticide (Supplementary Data, Figure S1). The CO_2_ propellant within the high-pressure cylinder created ~5.5 Megapascals (800 psi) of pressure at the two nozzles, and the VMD from the spray of this nozzle was ~18 µm, while the 10% and 90% volume diameters were 9 µm and 34 µm [16].

The second aerosol insecticide was a combination of Pyrocide 100^®^ (795 mL, MGK, Minneapolis, MN, USA) and Diacon II (16 mL, Wellmark, Schaumburg, IL, USA). Pyrocide 100 contains 1.0% pyrethrins (AI), 2.0% piperonyl butoxide (synergist), and 3.0% N-Octyl bicycloheptene dicarboximide (synergist). Diacon II contains 33.6% (s)-methoprene (AI) and 66.4% other ingredients (Central Life Sciences, Schaumberg, IL, USA). Hereafter, this aerosol insecticide will be referred to as Pyrocide. This insecticide was applied using an electric, hand-held fogger (Micro-Jet ULV Fogger 7401, Fogmaster, Deerfield Beach, FL, USA). The Micro-Jet fogger is designed with a high-airflow venturi nozzle (Supplementary Data, Figure S2). A fan supplies 1.1 m^3^/s (40 cfm) of airflow through the nozzle, which suctions liquid into the nozzle and atomizes it into small drops via air turbulence. The fan also provides a jet of air to dispense fog. The liquid flow was set at 280 mL/min and released at a height of ~1 m. The Micro-Jet 7401 produced larger-sized droplets with a VMD of ~58 µm, while 10% of the spray was ~20 µm or less. 

Each formulation described contains pyrethins and an insect growth regulator (pyriproxyfen or methoprene). These formulations were chosen to provide differences in droplet size and distribution patterns to shed light on aerosol insecticide dispersion and deposition patterns and their correlated effect on *T. confusum,* an indicator stored product insect species, and how application location influences these parameters. 

### 2.2. Aerosol Application Locations 

Aerosol applications were made from one of three locations (south, east, or northwest) or evenly split among the three locations. All aerosol applications were made with a commercial applicator at the labeled dosage rate based on the volume of the room. The dosages were ~900 g/trial for Turbocide and 801 mL of liquid per trial for Pyrocide. The 16 aerosol trials were conducted over a 3-day period as described previously [5]. The cylinderized Turbocide was applied during trials 1–8, while the hand-held sprayer containing the Pyrocide was applied during trials 9–16. Each aerosol was applied at a specific spray release location (or evenly split among all three locations), which took ~3 min to dispense, and then the room was left undisturbed for 60 min to allow the droplets to settle. During this holding period, the building air system was turned off and the room air vents were closed. 

Application 1 was from the south location. Application 2 was from the east location. Application 3 was from the northwest location. The final combination included spray portions from each of the three primary locations, which started at the northwest location, moved to the east location, and finished at the south location. A total of 16 trials were conducted (2 aerosol insecticide systems × 4 application locations (south, east, northwest, multi-location) × 2 replicates). The dispensed aerosol per trial was monitored over 60 min at 9 locations throughout the mill floor. Between trials, the air vents were opened, and the room was ventilated with fresh air until air droplet counts within the room returned to background levels (~15 min). Control bioassay arenas, described previously by Scheff et al. [5,15], were held on a separate floor of the mill where experienced similar environmental conditions and handling, but without exposure to aerosol treatments. No adult mortality or extraneous effects were observed or reported in the control arenas. In addition, the aerosol monitoring equipment collected ~10 min of baseline data prior to spray release. 

### 2.3. Measurement of Aerosol Droplets

Nine Aerodynamic Particle Sizer spectrometers (APS) (model 3321, TSI, Shoreview, MN, USA) were strategically placed throughout the flour mill to measure aerosol droplet numbers and mass concentrations during each trial. Some units were in unobstructed areas, depending on the application location, while others were behind or under equipment (Figure 1 and Figure S3). The APS units are designed to measure droplets 0.5 to 20 µm in diameter and, for 10 µm droplets, to count over 20,000 droplets per second with less than 10% error (Supplementary Table S1). Each APS instrument was connected to a laptop computer running Aerosol Instrument Management (v. 9.0) software (TSI Inc., Minneapolis, MN, USA) to record the data collected during a trial. After each trial, the software was paused and data was saved. 

Overall, each trial consisted of ~80 min of recorded data (~5 min pre-trial; 60 min treatment period; and ~15 min ventilation time) consisting of over 240 sampling intervals in a data profile. Starting points for the treatment periods in the aerosol data profiles were determined by detecting the first data points with mass concentration values above a threshold level of 0.5 mg/m^3^. The data collected during the pre-trial and ventilation periods were deleted from the aerosol data profiles used for analysis, resulting in 180 lines of observations or 60 min of data per profile.

### 2.4. Mass Concentration Index (MCI)

Each APS unit counted droplets over 20 sec intervals and stored the count values in a series of 52 “bins”, representing different ranges of droplet diameters between 0.5 and 20 µm. There were 32 size bins for drops from 0.5 to 5 µm and 20 size bins for drops from 5.0 to 20 µm. The droplet count data were converted to mass concentration using droplet density and volume for each size bin, and an instantaneous total mass concentration value was determined by summing the mass concentration values from all 52 droplet size bins for each sampling interval. The total mass concentration values were then accumulated over the full 60 min treatment period, and this grand summation was called the mass concentration index (MCI). The mean (+/− standard error of the mean) MCI values were tabulated for each treatment combination, replicate, and APS unit location. For each insecticide treatment, the MCI was analyzed in a two-way ANOVA using PROC MIXED in SAS/STAT software (v. 9.4, SAS Institute, Cary, NC, USA). This initial analysis revealed significant variation for aerosol application position, APS location, and the interaction between the two factors. The data on MCI were analyzed separately with the aerosol release position and with the unit location. Means were compared using Tukey–Kramer mean separations. 

### 2.5. Deposition Index (Dep.Idx)

The APS instrument collects and measures air samples ~15 cm above the floor and therefore does not directly measure insecticide deposition. Each APS draws air continuously through the sampling port at 5 L/min, and then a 1 L/min subsampled is passed through the laser optical counter system to count the droplets collected. The unit of measure normally provided with the APS system is mg/m^3^ and represents a concentration of droplets in a large volume of air. A new metric was developed to estimate the mass of droplets deposited on a given area of floor, hereby termed the *Deposition Index* (Dep.Idx), contains the units of ‘g/m^2^’. The Dep.Idx was determined using the mass of droplets collected with the APS unit during the 60 min trial and dividing by the approximate cross-sectional area of the APS air sampling tube (~32 mm^2^). The mass (mg) of the droplets for the trial was proportional to the MCI value by dividing by a conversion factor of 3000. A second mathematical calculation was required to convert the ‘mg/mm^2^’ unit to the ‘g/m^2^’ unit. The Dep.Idx did not include the small drops that tend to drift. The Dep.Idx was computed using the APS MCI data for the 8–20 µm size range of droplets because those droplets settle to the floor within 30–60 min. Also, the 8–20 µm size range roughly corresponded to medium and large droplet sizes reported in earlier research by Arthur et al. [12,17]. As a reference mass balance calculation, the mass of the Turbocide applied per trial (900 g) was divided by the approximate milling floor area (~21 m × 14 m) or 900 g/300 m^2^ or ~3 g/m^2^. To visualize spatial variation in estimated deposition, contour plots of Dep.Idx values were generated using Kriging with Surfer (v. 25) software (Golden Software, Golden, CO, USA). Also, the percentage area between contour lines was determined using the image processing routine in ImageJ (http://imagej.nih.gov/ij, v1.53t). For low depositions and low efficacy, the Dep.Idx ranged from 0 to 2 g/m^2^; for medium deposition, the Dep.Idx ranged from 2 to 4 g/m^2^; and for high deposition, the Dep.Idx ranged from 4 g/m^2^ to higher. These zones of deposition were created to correspond with low and high levels of insect efficacy. 

### 2.6. Relationship between Aerosol Droplets and Insecticide Efficacy

We evaluated the relationship between aerosol droplet measurements discussed above and insecticide efficacy data reported in Scheff et al. [6,15]. Full details on the methodologies and results can be found in those earlier manuscripts. The direct effects of the pyrethrin portion of the aerosol insecticide on adults were assessed by exposing 10 adult *T. confusum* in bioassay arenas during each spray treatment. Bioassay arenas were placed near each of the nine APS units. Adult efficacy was determined by classifying each adult as either live, knocked-down/affected, or dead. For analysis in this paper, the efficacy data reported by Scheff et al. [6] were converted into an adult efficacy index (E.I.) based on the one developed by Campbell et al. [9]. Since this experiment used 10 adults per dish, the index values ranged from a low of 1 (all alive) to a high value of 66 (all dead), and values of 56 and above had all adults either affected or dead. 

Residual efficacy due to the IGR was evaluated by placing empty bioassay arenas near each APS instrument during treatments and, after 2 weeks, adding five larvae to the arenas and monitoring their development. An efficacy index was used to characterize the impacts of the insecticide [15], with the lowest efficacy having a value of 1, corresponding to all five larvae developing into normal adults, and the highest efficacy having a value of 21, which was assigned when no larvae successfully developed to the adult stage. To help identify the trends between the Dep.Idx and efficacy, the replicates were averaged. The average adult E.I. and the average larvae E.I. were compared to the average Dep.Idx per APS and per spray treatment. Each plot contained 36 observations, which include multipoint, south, east, and northwest spray locations for the 9 APS units. Data correlations were determined for the plots using Pearson’s correlation function. 

## 3. Results

### 3.1. Instantaneous Mass Concentrations 

**Figure 2:**
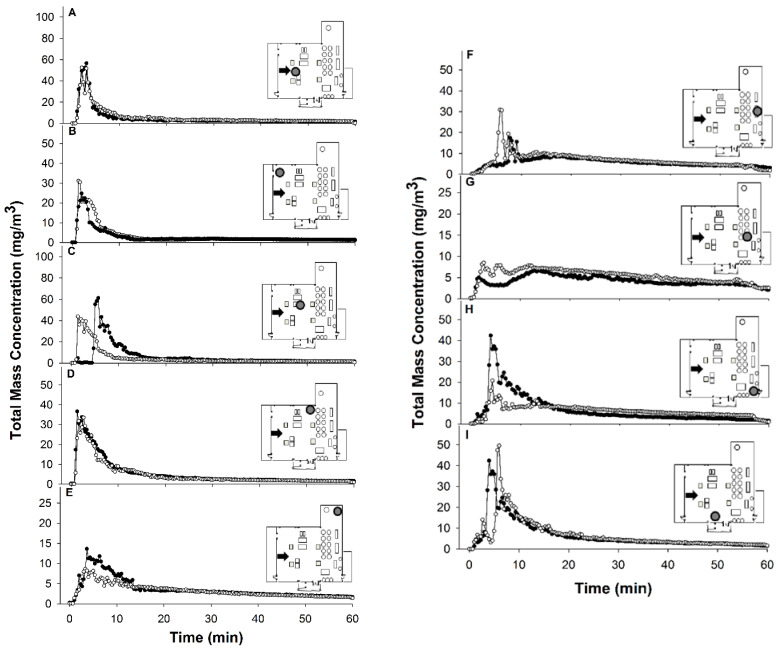
APS data after Turbocide treatments with the high-pressure cylinder. Nine subplots (**A**–**I**) display total mass concentration (mg/m^3^) values per APS unit during 60 min exposure time. The inset schematic shows the spray location (black arrow) and APS position corresponding to that subplot (gray circles). Two replicate treatments are shown in each plot: trial 1 (black dots) and trial 2 (white circles).

**Figure 3:**
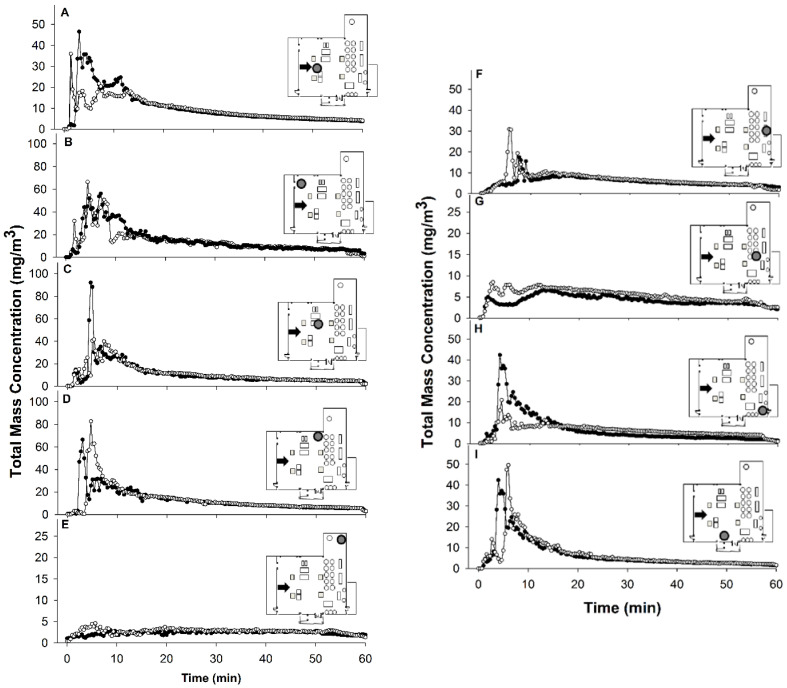
APS data after Pyrocide treatments with hand-held sprayer. Nine subplots (**A**–**I**) display total mass concentration (mg/m^3^) values per APS unit during 60 min exposure timeThe inset schematic shows the spray location (black arrow) and APS position corresponding to that subplot (gray circles). Two replicate treatments are shown in each plot: trial 1 (black dots) and trial 2 (white circles).

In general, the mass concentration values increased rapidly at the start of the treatment, as expected with the release of the aerosol insecticide droplets, followed by a rapid decrease within the first 10 min, and then by a long, gradual decrease to a baseline concentration during the remaining 50 min of each trial (Figure 2, Figure 3 and Figure S4a–h). The period of high mass concentration occurred within the first 10 min of the treatment, regardless of the location of the APS unit. However, the timing and size of the peak in mass concentration varied with location, with more distant (further from the application point) and obstructed locations generally having smaller peaks. These findings suggest that shorter holding periods, i.e., ~40 min before re-entry into treated rooms, would likely provide similar efficacy since most of the droplets had settled within the first half of our 1 h treatment periods. 

### 3.2. Mass Concentration Index (MCI) 

#### 3.2.1. Spray Locations vs. Spray Systems

**Figure 4:**
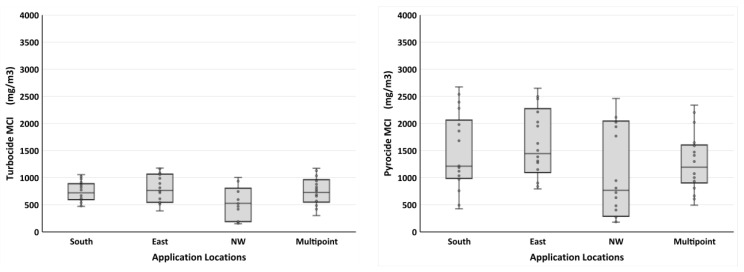
Box plots showing mass concentration index (MCI) data. Turbocide (**left**) and Pyrocide (**right**) were applied from either the south side, east side, northwest (NW) corner, or split equally among three locations (multipoint). Each box plot represents the MCI values from two trials and nine APS units (*n* = 18).

Spray application location did not significantly impact mean MCI for either Turbocide (ANOVA: *F* = 0.133; df = 3,68; and *p* = 0.940) or Pyrocide (*F* = 0.347; df = 3,68; and *p* = 0.791), although the northwest corner location tended to have more locations with lower MCI values (Figure 4). For Turbocide, the median MCI values for the south, east, and multipoint application locations were similar: 721, 766, and 728 mg/m^3^, respectively. However, the northwest application location had a median MCI of 527 mg/m^3^. For Pyrocide, the median MCI values for the south, east, and multipoint application locations were 1212, 1445, and 1194 mg/m^3^, respectively. The northwest application location had a median MCI value of 878 mg/m^3^.

#### 3.2.2. Spatial Variation of Turbocide Treatments

The Turbocide MCI values for each application location treatment were compared among APS instrument positions (Table 1), and differences in the MCI appeared to be impacted by distance from the application location and level of obstruction. The mean MCI per spray location were 757, 739, 800, and 717 mg/m^3^ for the multipoint, south, each, and northwest applications, respectively, and these means were significantly influenced by application location (*F* = 4.0; df = 3,36; and *p* < 0.014). The aerosol monitoring instruments were statistically different (*F* = 94.0; df = 8,36; and *p* < 0.0001), and the interaction between application location and APS position was also significant (*F* = 53.1; df = 24,36; and *p* < 0.0001). APS positions 1, 3, and 4 had mean MCI values greater than 900 mg/m^3^ and were more centrally in the mill, in an unobstructed area, and proximate to both the south and east application locations. In contrast, APS position 7, which was underneath equipment and near the north wall, had MCI values below 500 mg/m^3^ from the south and east treatments. APS position 2 was in the southwest corner of the room and reported lower MCI values from all the treatments, likely because it was behind the direction of spray for south and east treatments and far from the northeast application location. The northwest corner application location resulted in the widest range of Turbocide MCI values, ranging from over 2500 mg/m^3^ at APS position 5, which was next to the application point, to below 200 mg/m^3^ at APS positions 1, 2, and 9, which were farthest from the northwest corner.

#### 3.2.3. Spatial Variation of Pyrocide Treatments

The Pyrocide MCI values for each application position treatment were compared among APS instrument locations (Table 2), and differences in the MCI, like Turbocide, appeared to be impacted by distance from application location and level of obstruction. The Pyrocide mean MCI values per spray location were 1286, 1490, 1630, and 1593 mg/m^3^ for the multipoint, south, each, and northwest applications, respectively, and were significantly influenced by application location (*F* = 11.8; df = 3,36; and *p <* 0.0001), APS location (*F* = 61.3; df = 8,36; and *p* < 0.0001), and the interaction between the two factors (*F* = 60.5; df = 24,36; and *p* < 0.0001). The multipoint, south, and east spray treatments covered the middle of the room well and had the highest MCI values, from 1400 to 2600 mg/m^3^, for APS units 1, 3, and 4. The APS positions in the corners tended to have lower MCI values, such as APS positions 2, 5, and 9, with values that tended to be below 1000 mg/m^3^. When the spray was released from the northwest corner, APS unit 5 nearest that application location detected extremely high MCI values around 5658 mg/m^3^, but other APS positions tended to have lower MCI values when compared to other application locations.

#### 3.2.4. Relationship between Aerosol Droplets and Insecticide Efficacy

**Figure 5:**
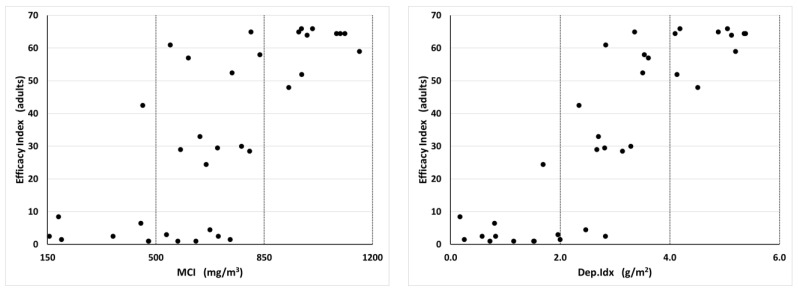
Relationship between Turbocide aerosol insecticide MCI and Dep.Idx values and insecticide efficacy index values for adults, which is a measure of the efficacy of the pyrethrins and synergists portion of the insecticide treatment. Each plot contains 36 data points, representing four different spray release locations (south, east, northwest, and multiple positions) and nine APS positions within each treatment. The Pearson correlation coefficient for the Turbocide versus the adult efficacy was 0.749 with MCI and 0.872 with Dep.Idx.

**Figure 6:**
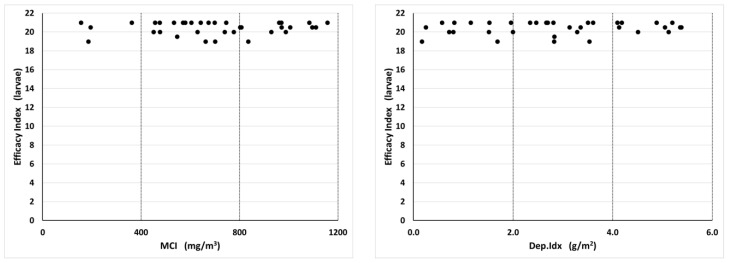
Relationship between Turbocide aerosol insecticide MCI and Dep.Idx values and insecticide efficacy index values for larvae, which is a measure of residual effects of the IGR on development 2 weeks after treatment. Each plot contains 36 data points, representing four different spray release locations (south, east, northwest, and multiple positions) and nine APS positions within each treatment. The Pearson correlation coefficient for the Turbocide versus the larval efficacy was 0.178 with MCI and 0.146 with Dep.Idx.

The two insecticide treatments exhibited different relationships between the MCI and efficacy against both adults and larvae. Adult efficacy values provide a measure of the effects of the pyrethrin portion of the aerosol formulation when the adults are directly exposed to it during the treatment. The larval efficacy values measure the residual effects of the IGR portion of the aerosol formulation. The adult and larval efficacy indices were compared with APS measurements, which included all four spray scenarios and all nine APS units. With Turbocide, the adult efficacy index increased with increasing MCI and Dep.Idx values in a sigmoid pattern with a transition between low and high efficacy between 500 and 850 mg/m^3^ for the MCI and 2 and 4 mg/m^2^ for the Dep.Idx (Figure 5). Below this range, efficacy was low and relatively stable (efficacy values ≤10 indicate 7 or more of the 10 adults appearing unaffected by the insecticide). Above this range, efficacy was high and relatively stable (efficacy values ≥46 indicate only 0–1 of the 10 adults appearing unaffected by the insecticide). In contrast, the larval efficacy index was high at all measured values of the MCI and Dep.Idx (Figure 6). All larvae efficacy index values were over 16, which indicates that no larvae developed to the adult stage in the bioassays.

**Figure 7:**
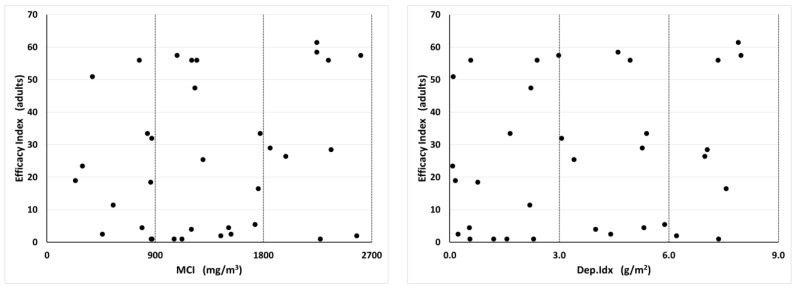
Relationship between Pyrocide aerosol insecticide MCI and Dep.Idx values and insecticide efficacy index values for adults, which is a measure of the efficacy of the pyrethrins and synergists portion of the insecticide treatment. Each plot contains 36 data points, representing four different spray release locations (south, east, northwest, and multiple positions) and nine APS positions within each treatment. The Pearson correlation coefficient for the Pyrocide versus the adult efficacy was −0.028 with MCI and −0.082 with Dep.Idx.

**Figure 8:**
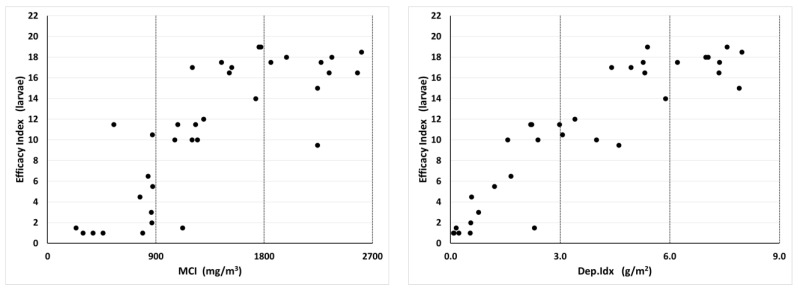
Relationship between Pyrocide aerosol insecticide MCI and Dep.Idx values and insecticide efficacy index values for larvae, which is a measure of residual effects of the IGR on development 2 weeks after treatment. Each plot contains 36 data points, representing four different spray release locations (south, east, northwest, and multiple positions) and nine APS locations within each treatment. The Pearson correlation coefficient for the Pyrocide versus the larval efficacy was 0.670 with MCI and 0.906 with Dep.Idx.

For the Pyrocide applications, there was no apparent relationship between MCI and Dep.Idx values and insecticide efficacy against adults, with efficacy levels being highly variable across the whole range of measured droplet values (Figure 7). In contrast, for insecticide efficacy against larvae, there was a trend for efficacy to increase with the increasing MCI or Dep.Idx before plateauing above 1800 mg/m^3^ or 6 mg/m^2^, respectively (Figure 8). The relationship between MCI and efficacy appeared to be more sigmoid, while the relationship between the Dep.Idx and efficacy was more linear.

#### 3.2.5. Spatial Pattern in Aerosol Droplets

**Figure 9:**
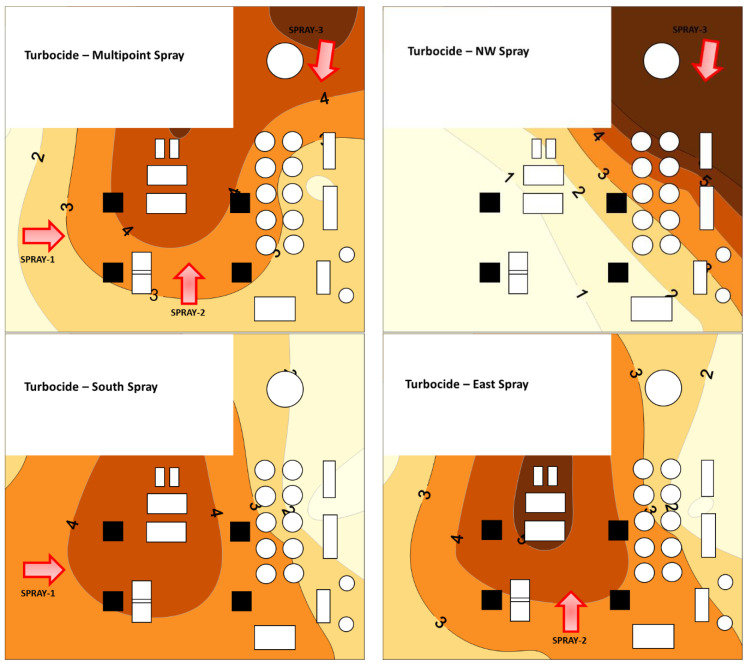
Contour maps showing estimated Turbocide insecticide deposition (Dep.Idx, g/m^2^) in a pilot-scale flour mill when applied from three locations or equally split among the three locations in varied spray positions. Increasingly dark contour zones represent areas estimated to have increasing Dep.Idx values (from <1 to >5 g/m^2^).

**Figure 10:**
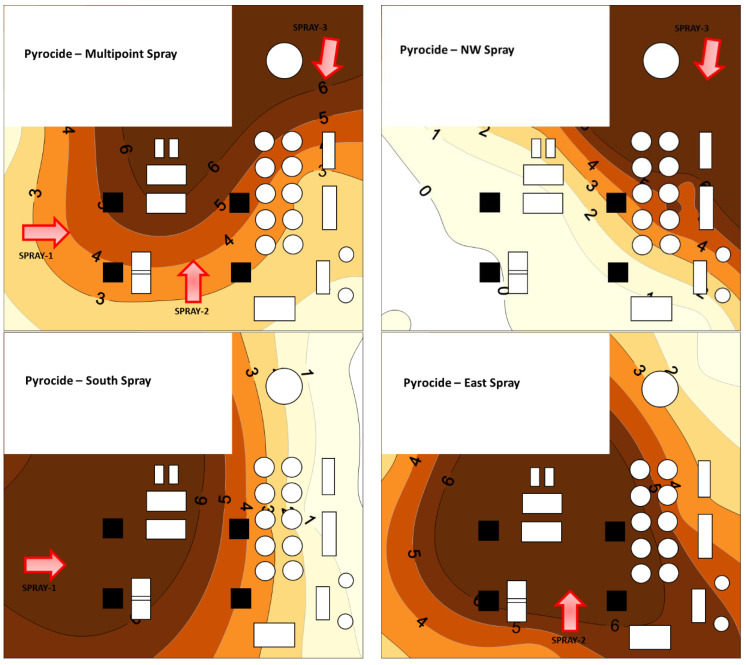
Contour maps showing estimated Pyrocide insecticide deposition (Dep.Idx, g/m^2^) in a pilot-scale flour mill when applied from three locations or equally split among the three locations in varied spray positions. Increasingly dark contour zones represent areas estimated to have increasing Dep.Idx values (from <1 to >6 g/m^2^).

Application position impacted the spatial pattern of the predicted chemical deposition within the mill (Figure 9 and Figure 10). For Turbocide, multipoint application and east side application resulted in 7% and 13% of the floor area having a low-zone Dep.Idx, as shown in Table 3. The northwest application resulted in 54% of the floor area being a low-zone Dep.Idx. A high-zone Dep.Idx was only 24–32% for all application scenarios. For Turbocide, three application locations had ~60% of the floor area in the medium zone, and they were the multipoint, east, and south side applications. For Turbocide, Dep.Idx in the high zone was associated with high efficacy against adults, but residual efficacy against larvae was high across all measured the Dep.Idx, so all portions of the mill were predicted to have good residual efficacy for larvae.

Pyrocide treatments had similar general trends to Turbocide, with the multipoint application and east side application having smaller zones with low deposition, with 5% and 8% of the floor area having a low Dep.Idx (Figure 10 and Table 3). The northwest application and the east side application resulted in 55% and 25% of the floor area with a low Dep.Idx, respectively. For Pyrocide, more floor area was in the high zone with ~50%, 60%, and 70% areas for multipoint, south side, and east side applications, respectively. For Pyrocide, the Dep.Idx in the high zone was associated with high residual efficacy against larvae. However, efficacy against adults was highly variable across all measured levels of the Dep.Idx, so all portions of the mill were expected to have random adult efficacy.

## 4. Discussion

In this study, we analyzed spatial and temporal patterns in droplet size distributions that occur during aerosol insecticide applications inside food facilities, developed a method to quantify these patterns, and explored the relationships between aerosol droplet size measurements and efficacy against insects. This study bridges the gap between a prior study that evaluated relationships between droplet size and insect efficacy conducted using vertical flow chambers [11,12,16,17] and spatial patterns in efficacy detected using bioassay insects in commercial applications [5,9,15,18,19]. Prior work relating aerosol droplets to insecticide efficacy within food facilities has been limited. Kharel et al. [20] studied the effect of pyrethrin applied in test sheds with a hand-held fogger on bioassays of *Tribolium castaneum* (Herbst) and *Tribolium confusum* on varied surface types with and without residual flour. Residual flour increased insect survival significantly. Scheff et al. [3] evaluated a single pyrethrin and IGR aerosol treatment in a commercial rice mill and used insect bioassays, finding that adult *T. confusum* mortality was greatest nearest the application points, but that efficacy levels were highly variable and recovery post-exposure was high at many locations. Residual efficacy against larvae was also variable but tended to be greater than the efficacy against adults. The IGR has greater activity at lower concentrations than the pyrethrin portion of the formulation. Aerosol droplets were also measured in this study using five APS instruments, and the mass concentration and deposition of aerosol droplets were found to be variable among locations, with a pattern that suggests more material is released earlier in the treatment than later in the treatment. Arthur et al. [8] evaluated aerosol droplet distribution at three locations along a transect on a different floor of the same mill used in this study. They found that droplet concentration and calculated deposition of two different aerosols decreased with increasing distance from the spray release location. Insect recoveries in bioassays also increased with increasing distances from application locations. And the measurements of droplet concentration around obstructed regions were reduced from those in regions with an open floor. This prior study showed that aerosol droplet size after being dispensed averaged ~10–12 µm, but that within ~20 min the larger droplets had settled out and average droplet sizes were only 4–6 µm. Our study employed more APS instruments than the previous study, and thus, we were better able to evaluate relationships between aerosol droplets and efficacy and could also evaluate how application location impacts spatial patterns in efficacy.

Differences in the distribution of aerosol droplet size ranges are likely to cause differences in efficacy observed in field studies. In flow chamber studies, where the droplet size distributions and concentrations can be carefully controlled, it has been demonstrated that droplet size contributed to efficacy more than concentration and that small droplets (2 µm) had lower efficacy than larger droplets (16 µm) [17]. This results from the smaller droplets tending to float with the air streams rather than impinging on the insects or the arena surfaces, and this effect in flow chambers has also been predicted using a modeling approach [16]. In commercial applications, unlike in flow chambers, aerosol droplets are released over a short period of time and allowed to disperse and settle over time, which can result in temporal and spatial variation in droplets in the air and deposited on surfaces. In our study, as in Arthur et al. [8], the droplet mass data, which were collected periodically and over 60 min, show a large spike in mass concentration soon after insecticide release, followed by a long tail consisting of a low concentration of primarily small-diameter droplets. The height and space of these spikes and their concentration varied with distance from the application point and the occurrence of physical features that could obstruct droplet movement. Here, we found considerable variation among locations in the droplet concentration and size distributions, which could ultimately impact the deposition of insecticide on surfaces. These impacts are shown in the spatial variation in mass concentration and deposition patterns among application locations.

A new method to calculate deposition that has been used in earlier studies was developed and is predicted to provide a more accurate estimation of the amount of insecticide being applied to a surface or an insect walking on that surface. The characteristics of an aerosol application in a food facility are difficult to summarize compared to those in a vertical flow chamber, given that application methods produce a range of droplet sizes. Also, the number of droplets of each size changes with time after the initiation of the application due to differences in the settling rates of droplets of different sizes. Additionally, the concentration and size distributions vary spatially within a room due to distance from the application point and physical features in the landscape that impact droplet movement. There are also limitations in assessing droplet characteristics due to the limitations of the measuring equipment itself, such as the limited range of sizes that can be accurately detected and quantified and the number of locations where measurements can be taken logistically. Ultimately, the amount of active ingredient deposited on a surface or insect within a food facility will affect efficacy. This is typically going to be a function of the droplet size, since larger droplets settle more quickly and travel shorter distances than smaller droplets.

There are different ways to estimate this potential deposition. Scheff et al. [3] estimated the deposition of an aerosol treatment of a rice mill via summation of real-time mass concentrations, or MCI, as provided using fundamental data from the APS instruments. However, deposition estimates based on the MCI do not take into consideration the rates of deposition of different droplet sizes. More precisely, small droplets that linger in the air continue to be counted using the APS instrument and inflate MCI values while the large- and medium-sized droplets have settled out. Here, we presented a method of deposition estimation, Dep.Idx, that may be more representative of the material that deposits on the floor since it considers the mass of the medium and larger droplets rather than including small droplets and uses the basic units of mass/area (g/m^2^). Also, the Dep.Idx estimate was similar to the overall chemical applied and the given floor area. For example, in our experiments, 900 g of Turbocide was applied per trial over a 21 m × 14 m floor, 900-g/300-m^2^ or ~3 g/m^2^. There may be other factors that affect the exact deposition, such as chemical evaporation, volatility, or deposition onto walls, ceilings, and other structures in the room. Additional test methods have been developed to chemically determine the deposition of IGR–methoprene, which could provide validation measurements of deposition in future testing [21].

There were inherent differences between both spray systems that were evident in aerosol droplet measurements, spatial patterns in deposition, and relationships with efficacy against insects. Although there are differences in formulation that could contribute to the differences in efficacy, the application method also differs, which could also impact the efficacy. Specifically, Turbocide was applied with a high-pressure cylinder that produces a droplet distribution with a VMD of ~18 μm, while Pyrocide was applied with a blower and nozzle system that produces droplets with a larger size distribution (VMD of ~50 μm) that were propelled over shorter distances [16]. However, in our study, Pyrocide had regions with higher deposition amounts than Turbocide, suggesting that the two products need to be applied differently to achieve desired levels of effectiveness across the whole space. However, caution must be exercised when comparing APS data from both systems, as APS units are only capable of counting droplets that are 20 µm or smaller. Some discrepancies could arise when trying to compare Turbocide to Pyrocide data because the APS collects portions of each spray distribution and not the entire size range of droplets that are being produced with each spray.

Our results show that relationships between an aerosol application system and efficacy can be developed, which will guide the development of more effective application strategies. However, our results suggest these relationships will be unique to a given formulation and application system. The lack of relationships between the MCI and efficacy observed in some cases could result from multiple reasons. First, the MCI/Dep.Idx measurements may not accurately represent actual deposition in bioassay arenas. For example, deposition could be underrepresented due to the occurrence of larger droplets that are outside the range of the APS instruments. Additionally, there may be greater variation in spray distribution, which would impact the relationship between efficacy and MCI/Dep.Inx. This is relevant because the bioassays were 10 to 20 cm away from the APS unit, and thus, MCI data and bioassay data are not perfectly matched throughout the entire facility. The MCI/Dep.Idx index may also not fully capture all the variables influencing the relationship between droplets in the air and deposition. Further research and analysis of these relationships is needed, but the variation in droplet predicted deposition does appear to be a useful measurement for determining the evenness of coverage and may be less prone to variation relative to the bioassay data. The two insecticide formulations and delivery methods resulted in differences in spatial patterns in droplet MCI/Dep.Idx. Consistently higher efficacy indices on both *T. confusum* larvae and adults with Turbocide than Pyrocide suggested that, in addition to delivery systems, the differences in formulations could be influential on efficacy. The results of this current field study are consistent with those using controlled experiments in vertical flow chambers, in that aerosol droplet size and residual deposition are important factors that affect the insecticidal efficacy of adults and larvae of stored product insects [11,12,13,16]. Future studies could be conducted to elucidate the influence of structural barriers more accurately inside flour mills to assess aerosol efficacy and how spray deposition patterns affect efficacy. In addition, the actual aerosol insecticide that is dispensed from an application system can also affect the resulting efficacy.

## Figures and Tables

**Table 1:** Turbocide aerosol treatment means mass concentration index (MCI, mg/m^3^) measured at nine APS positions throughout the mill floor. Turbocide aerosol was released from one of four application location (south, east, northwest, and multipoint) treatments (*n* = 2). The pooled SEM was 75.0. Means followed by same lowercase letter denote no significant difference between APS locations for each spray release position (columns) via Tukey’s HSD (α = 0.05.)

Multipoint	APS	South	APS	East	APS	NW	APS
1157 a	5	969 a	1	1110 a	4	2549 a	5
1005 ab	3	961 a	3	1094 a	3	971 b	7
988 ab	4	929 ab	4	1083 a	1	745 bc	6
807 bc	1	802 abc	8	835 b	8	642 cd	4
699 cd	6	701 bcd	6	776 bc	6	534 cd	8
674 cd	8	629 cd	5	739 bc	5	475 d	3
662 cd	7	604 cd	9	570 cd	2	195 e	2
457 de	9	579 cd	2	546 cd	9	185 e	1
361 e	2	476 d	7	451 d	7	155 e	9

**Table 2:** Pyrocide aerosol means mass concentration index (MCI, mg/m^3^) measured at nine APS positions throughout the mill floor. Pyrocide aerosol was released from one of four application location (south, east, northwest, and multipoint) treatments (*n* = 2). The pooled SEM was 138. Means followed by same lowercase letter denote no significant difference between APS locations for each spray release position via Tukey’s HSD (α = 0.05).

Multipoint	APS	South	APS	East	APS	NW	APS
2271 a	5	2607 a	1	2574 a	3	5658 a	5
1746 ab	4	2338 b	4	2361 ab	1	2242 b	7
1530 abc	1	1986 c	3	2242 ab	4	2114 b	4
1444 bc	3	1772 c	2	1728 bc	8	1854 bc	6
1243 bcd	7	1203 d	9	1509 cd	6	790 cd	3
1082 cd	6	1121 de	6	1297 cd	7	768 cd	8
871 cd	2	1056 de	8	1230 cd	2	378 d	1
833 cd	8	866 e	7	869 d	9	294 d	2
551 d	9	460 f	5	861 d	5	235 d	9

**Table 3:** Estimates of percent area of floor with low, medium, and high amounts of insecticide deposits. The three zones were separated as 0–2 g/m^2^ for low deposits, 2–4 g/m^2^ for medium deposits, and greater than 4 g/m^2^ for high deposits.

		Zones of Depositions (g/m^2^)		
Insecticide	Location	Low	Med	High
		0–2	2–4	>4
Turbocide	Multi	7%	61%	32%
Turbocide	East	13%	62%	25%
Turbocide	South	19%	58%	24%
Turbocide	NW	54%	17%	29%
				
Pyrocide	Multi	5%	46%	49%
Pyrocide	East	8%	22%	70%
Pyrocide	South	25%	16%	59%
Pyrocide	NW	55%	11%	34%

## Data Availability

Please request any particular data through the corresponding author. And public data will be added to the USDA NAL AG commons and data repository.

## Supplementary Materials

The following supporting information can be downloaded at https://www.mdpi.com/article/10.3390/insects14120914/s1. Figure S1 Photo of Turbocide fog being dispensed by commercial applicator at KSU experimental mill; Figure S2 Photos of 2018 spray trials with Microjet-7401 at USDA-ARS facility in College Station, TX.; Figure S3 Example of APS unit deployed at KSU experimental mill for measuring spray mass concentrations during 60 minute holding period; Figure S4a: Real time mass concentrations (mg/m^3^) of Turbocide aerosol particles detected by Aerodynamic Particle Sizers (APS) at nine locations after released from a high pressure cylinder in a flour mill. The aerosol was released at the north-west location (shown with a solid arrowhead) and detected by APS in each of the nine locations (shown with a large gray circle) in each inserted floor plan (A–I). In each graph, the two lines represent the mass concentrations obtained for 20-s intervals over a 60 min-exposure to the aerosol in trial 1 (shown with solid circles) and trial 2 (shown with open circles); Figure S4b: Real time mass concentrations (mg/m^3^) of Pyrocide aerosol particles detected by Aerodynamic Particle Sizers (APS) at nine locations after released from a portable hand-held fogger in a flour mill. The aerosol was released at the north-west location (shown with a solid arrowhead) and detected by APS in each of the nine locations (shown with a large gray circle) in each inserted floor plan (A–I). In each graph, the two lines represent the mass concentrations obtained for 20-s intervals over a 60 min-exposure to the aerosol in trial 1 (shown with solid circles) and trial 2 (shown with open circles); Figure S4c: Real time mass concentrations (mg/m^3^) of Turbocide aerosol particles detected by Aerodynamic Particle Sizers (APS) at nine locations after released from a high pressure cylinder in a flour mill. The aerosol was released at multiple locations (shown with a solid arrowhead) and detected by APS in each of the nine locations (shown with a large gray circle) in each inserted floor plan (A–I). In each graph, the two lines represent the mass concentrations obtained for 20-s intervals over a 60 min-exposure to the aerosol in trial 1 (shown with solid circles) and trial 2 (shown with open circles); Figure S4d: Real time mass concentrations (mg/m^3^) of Pyrocide aerosol particles detected by Aerodynamic Particle Sizers (APS) at nine locations after released from a hand-held portable fogger in a flour mill. The aerosol was released at multiple locations (shown with a solid arrowhead) and detected by APS in each of the nine locations (shown with a large gray circle) in each inserted floor plan (A–I). In each graph, the two lines represent the mass concentrations obtained for 20-s intervals over a 60 min-exposure to the aerosol in trial 1 (shown with solid circles) and trial 2 (shown with open circles).; Figure S4e: Real time mass concentrations (mg/m^3^) of Turbocide aerosol particles detected by Aerodynamic Particle Sizers (APS) at nine locations after released from a high pressure cylinder in a flour mill. The aerosol was released at south location (shown with a solid arrowhead) and detected by APS in each of the nine locations (shown with a large gray circle) in each inserted floor plan (A–I). In each graph, the two lines represent the mass concentrations obtained for 20-s intervals over a 60 min-exposure to the aerosol in trial 1 (shown with solid circles) and trial 2 (shown with open circles); Figure S4f: Real time mass concentrations (mg/m^3^) of Pyrocide aerosol particles detected by Aerodynamic Particle Sizers (APS) at nine locations after released from a hand-held portable fogger in a flour mill. The aerosol was released at south location (shown with a solid arrowhead) and detected by APS in each of the nine locations (shown with a large gray circle) in each inserted floor plan (A–I). In each graph, the two lines represent the mass concentrations obtained for 20-s intervals over a 60 min-exposure to the aerosol in trial 1 (shown with solid circles) and trial 2 (shown with open circles); Figure S4g: Real time mass concentrations (mg/m^3^) of Turbocide aerosol particles detected by Aerodynamic Particle Sizers (APS) at nine locations after released from a high pressure cylinder in a flour mill. The aerosol was released at east location (shown with a solid arrowhead) and detected by APS in each of the nine locations (shown with a large gray circle) in each inserted floor plan (A–I). In each graph, the two lines represent the mass concentrations obtained for 20-s intervals over a 60 min-exposure to the aerosol in trial 1 (shown with solid circles) and trial 2 (shown with open circles); Figure S4h: Graphs showing real time mass concentrations of aerosol particles (mg/m^3^) obtained from nine Aerodynamic Particle Sizers (APS) at different locations in a flour mill exposed to Pyrocide aerosol released at east location (A–I). Each graph per APS unit with two lines represents mass concentrations obtained for 20 s intervals over a 60 minute-exposure to aerosol in trial 1 (black symbols) and trial 2 (white symbols). The inset in each graph is the floor plan of mill showing the location of APS unit (gray circle), and the location of Pyrocide aerosol released at east of the mill (black arrow) by using hand-held portable fogger; Table S1: Abbreviated table of APS mass concentration data for 52 drop size bins. Note 15 that columns between drop 1.11 μm and 14.9 μm are hidden. Note that rows from time 5.0 to time 55.0 are hidden.
