# Supplementary material for: Aerosolized Insecticide Spray Distributions and Relationships to Efficacy against Stored Product Pests
Source: Insects. 2023 Nov 28;14(12):914. doi: 10.3390/insects14120914 (PMC10744046; doi:10.3390/insects14120914)
Supplement: Supplementary file 1 [file insects-14-00914-s001.zip › insects-2662183-supplementary.pdf]

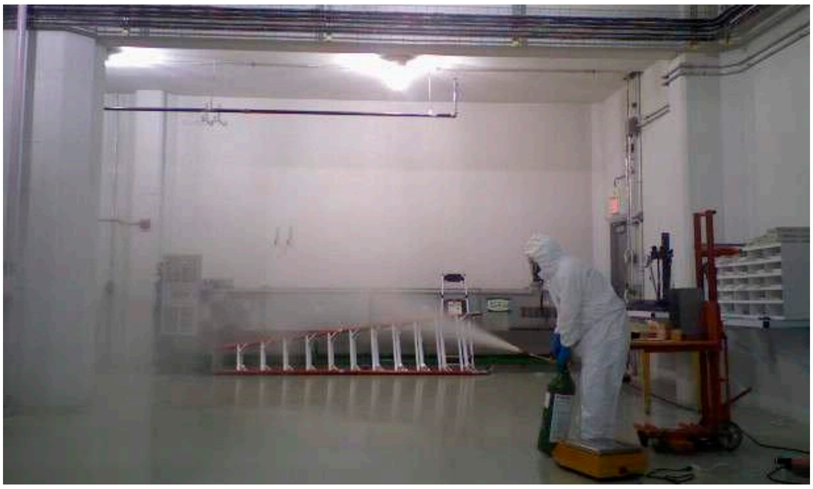

Figure S1. Photo of Turbocide fog being dispensed by commercial applicator at KSU experimental mill.

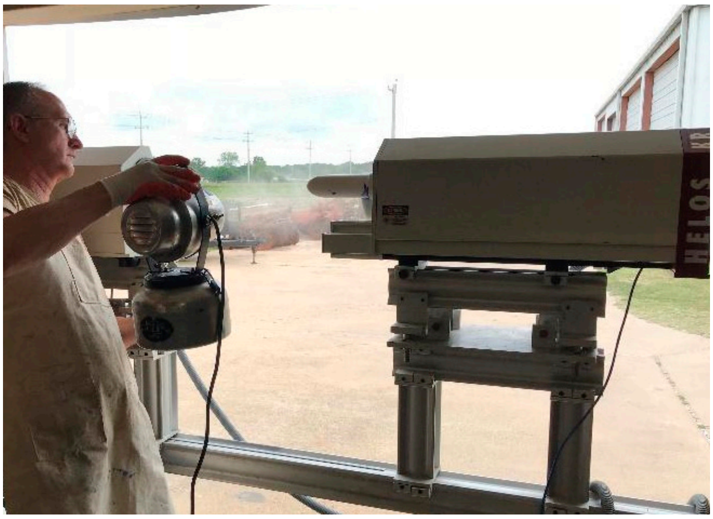

Figure S2. Photos of 2018 spray trials with Microjet-7401 at USDA-ARS facility in College Station, TX.

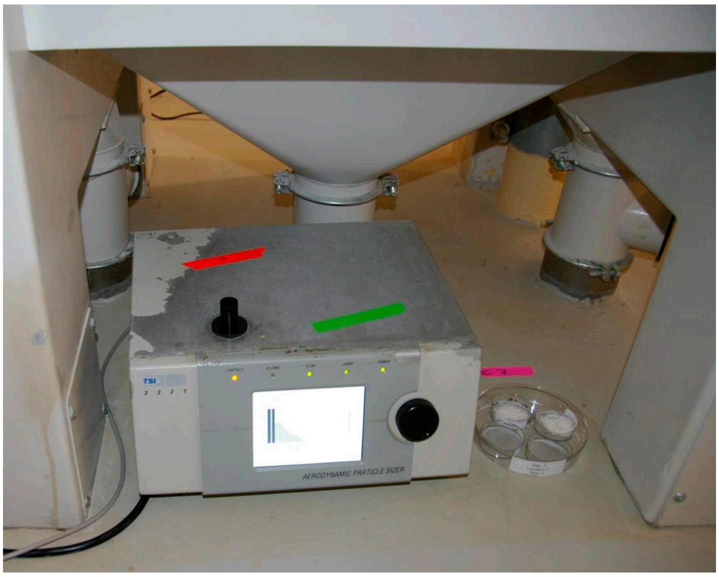

Figure S3. Example of APS unit deployed at KSU experimental mill for measuring spray mass concentrations during 60 minute holding period.

Table S1. Abbreviated table of APS mass concentration data for 52 drop size bins. Note that columns between drop 1.11um and 14.9um are hidden. Note that rows from time 5.0 to time 55.0 are hidden.

Sample File		C:\Users\Daniel.Brabec\Desktop\APS2016 Aug04 Thur\ksumill2016-11\Trial01-11.A21																				
Sample Time		20																				
Density		0.82																				
Stokes Correction		off																				
Lower Channel Bound		0.5																				
Upper Channel Bound		20.5			1	2	3	4	5	6	7	8	9	10	11	12		48	49	50	51	52
Sample #	Date	Start Time	Time (min)	ynamic Dia	<0.523	0.54	0.58	0.63	0.67	0.72	0.78	0.84	0.90	0.97	1.04	1.11		14.9	16.0	17.2	18.4	19.8
41	8/2/2016	10:05:22	0.0	dM	0.000	0.000	0.000	0.000	0.000	0.000	0.000	0.000	0.000	0.000	0.000	0.000		0.000	0.000	0.000	0.000	0.000
42	8/2/2016	10:05:42	0.3	dM	0.000	0.000	0.000	0.000	0.000	0.000	0.000	0.000	0.000	0.000	0.000	0.000		0.000	0.000	0.000	0.000	0.000
43	8/2/2016	10:06:02	0.7	dM	0.000	0.000	0.000	0.000	0.000	0.000	0.000	0.000	0.000	0.000	0.000	0.000		0.000	0.000	0.000	0.000	0.000
44	8/2/2016	10:06:22	1.0	dM	0.005	0.002	0.003	0.003	0.004	0.004	0.005	0.006	0.007	0.008	0.008	0.008		0.370	0.318	0.630	1.326	2.670
45	8/2/2016	10:06:42	1.3	dM	0.002	0.001	0.001	0.001	0.001	0.001	0.002	0.002	0.002	0.002	0.003	0.003		0.080	0.099	0.149	0.076	0.297
46	8/2/2016	10:07:02	1.7	dM	0.009	0.004	0.004	0.005	0.006	0.008	0.010	0.011	0.013	0.014	0.015	0.016		0.466	0.699	0.622	0.630	0.903
47	8/2/2016	10:07:22	2.0	dM	0.015	0.006	0.007	0.009	0.011	0.014	0.016	0.019	0.022	0.024	0.026	0.027		1.120	1.193	0.998	1.043	1.605
48	8/2/2016	10:07:42	2.3	dM	0.005	0.002	0.002	0.003	0.003	0.004	0.005	0.005	0.006	0.007	0.007	0.008		0.552	0.727	0.788	0.359	0.499
49	8/2/2016	10:08:02	2.7	dM	0.004	0.001	0.002	0.002	0.002	0.003	0.004	0.004	0.005	0.006	0.006	0.007		0.188	0.226	0.140	0.098	0.067
50	8/2/2016	10:08:22	3.0	dM	0.004	0.001	0.002	0.002	0.002	0.003	0.004	0.005	0.005	0.006	0.006	0.007		0.114	0.141	0.149	0.054	0.040
51	8/2/2016	10:08:42	3.3	dM	0.003	0.001	0.001	0.001	0.002	0.002	0.003	0.003	0.004	0.004	0.005	0.005		0.074	0.120	0.035	0.011	0.027
52	8/2/2016	10:09:02	3.7	dM	0.006	0.002	0.002	0.003	0.004	0.005	0.006	0.007	0.008	0.009	0.009	0.010		0.136	0.078	0.044	0.033	0.013
53	8/2/2016	10:09:22	4.0	dM	0.009	0.003	0.003	0.004	0.005	0.006	0.008	0.010	0.011	0.012	0.012	0.013		0.455	0.692	0.447	0.380	0.823
54	8/2/2016	10:09:42	4.3	dM	0.015	0.004	0.005	0.006	0.008	0.010	0.013	0.016	0.018	0.019	0.020	0.022		0.938	1.051	0.753	1.174	2.050
55	8/2/2016	10:10:02	4.7	dM	0.031	0.010	0.011	0.014	0.017	0.022	0.027	0.032	0.037	0.041	0.045	0.049		1.575	1.383	1.856	3.618	3.412
56	8/2/2016	10:10:22	5.0	dM	0.032	0.010	0.011	0.014	0.017	0.022	0.027	0.033	0.038	0.041	0.045	0.050		0.950	0.847	0.762	0.663	0.782
206	8/2/2016	11:00:22	55.0	dM	0.014	0.004	0.005	0.006	0.007	0.010	0.012	0.015	0.017	0.018	0.019	0.021		0.000	0.007	0.000	0.000	0.000
207	8/2/2016	11:00:42	55.3	dM	0.014	0.004	0.005	0.006	0.007	0.010	0.012	0.015	0.017	0.019	0.019	0.021		0.000	0.000	0.000	0.000	0.000
208	8/2/2016	11:01:02	55.7	dM	0.014	0.004	0.005	0.006	0.007	0.010	0.012	0.015	0.017	0.019	0.020	0.020		0.000	0.000	0.000	0.000	0.000
209	8/2/2016	11:01:22	56.0	dM	0.014	0.004	0.005	0.006	0.007	0.010	0.012	0.015	0.017	0.018	0.019	0.021		0.000	0.000	0.000	0.000	0.000
210	8/2/2016	11:01:42	56.3	dM	0.014	0.004	0.005	0.006	0.007	0.010	0.012	0.015	0.017	0.018	0.019	0.020		0.000	0.000	0.000	0.000	0.000
211	8/2/2016	11:02:02	56.7	dM	0.014	0.004	0.005	0.006	0.007	0.009	0.012	0.015	0.017	0.018	0.019	0.020		0.000	0.000	0.000	0.000	0.000
212	8/2/2016	11:02:22	57.0	dM	0.014	0.004	0.005	0.006	0.008	0.010	0.012	0.015	0.017	0.019	0.019	0.021		0.000	0.000	0.000	0.000	0.000
213	8/2/2016	11:02:42	57.3	dM	0.014	0.004	0.005	0.006	0.007	0.010	0.012	0.015	0.017	0.018	0.019	0.020		0.000	0.000	0.000	0.000	0.000
214	8/2/2016	11:03:02	57.7	dM	0.014	0.004	0.005	0.006	0.008	0.010	0.012	0.015	0.017	0.018	0.019	0.021		0.000	0.000	0.000	0.000	0.000
215	8/2/2016	11:03:22	58.0	dM	0.012	0.004	0.004	0.005	0.007	0.009	0.011	0.014	0.016	0.016	0.018	0.018		0.000	0.000	0.000	0.000	0.000
216	8/2/2016	11:03:42	58.3	dM	0.012	0.004	0.004	0.005	0.006	0.008	0.011	0.013	0.015	0.016	0.017	0.018		0.000	0.000	0.000	0.000	0.000
217	8/2/2016	11:04:02	58.7	dM	0.007	0.002	0.003	0.003	0.004	0.005	0.007	0.008	0.009	0.010	0.011	0.011		0.000	0.000	0.000	0.000	0.000
218	8/2/2016	11:04:22	59.0	dM	0.006	0.002	0.002	0.003	0.003	0.005	0.006	0.007	0.008	0.009	0.009	0.010		0.000	0.000	0.000	0.000	0.000
219	8/2/2016	11:04:42	59.3	dM	0.007	0.002	0.002	0.003	0.004	0.005	0.006	0.007	0.008	0.009	0.009	0.010		0.000	0.000	0.000	0.000	0.000
220	8/2/2016	11:05:02	59.7	dM	0.006	0.002	0.002	0.003	0.003	0.004	0.005	0.007	0.008	0.008	0.009	0.009		0.000	0.000	0.000	0.000	0.000
221	8/2/2016	11:05:22	60.0	dM	0.006	0.002	0.002	0.003	0.004	0.004	0.006	0.007	0.008	0.009	0.009	0.010		0.000	0.000	0.000	0.000	0.000

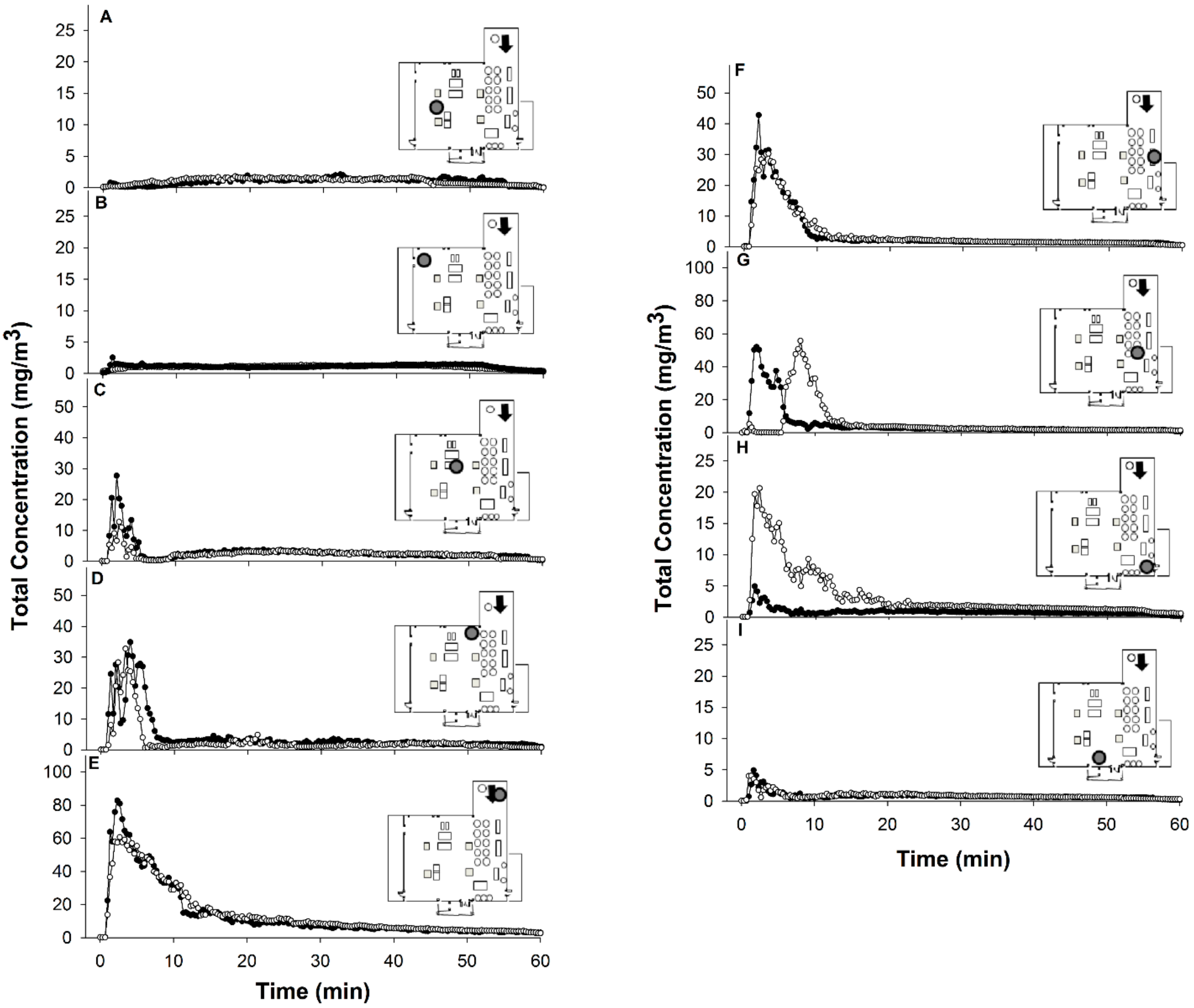

Figure S4a. Real time mass concentrations (mg/m^3) of **Turbocid**e aerosol particles detected by Aerodynamic Particle Sizers (APS) at nine locations after released from a high pressure cylinder in a flour mill. The aerosol was released at the **north-west** location (shown with a solid arrowhead) and detected by APS in each of the nine locations (shown with a large gray circle) in each inserted floor plan (A-I). In each graph, the two lines represent the mass concentrations obtained for 20-s intervals over a 60 min-exposure to the aerosol in trial 1 (shown with solid circles) and trial 2 (shown with open circles)

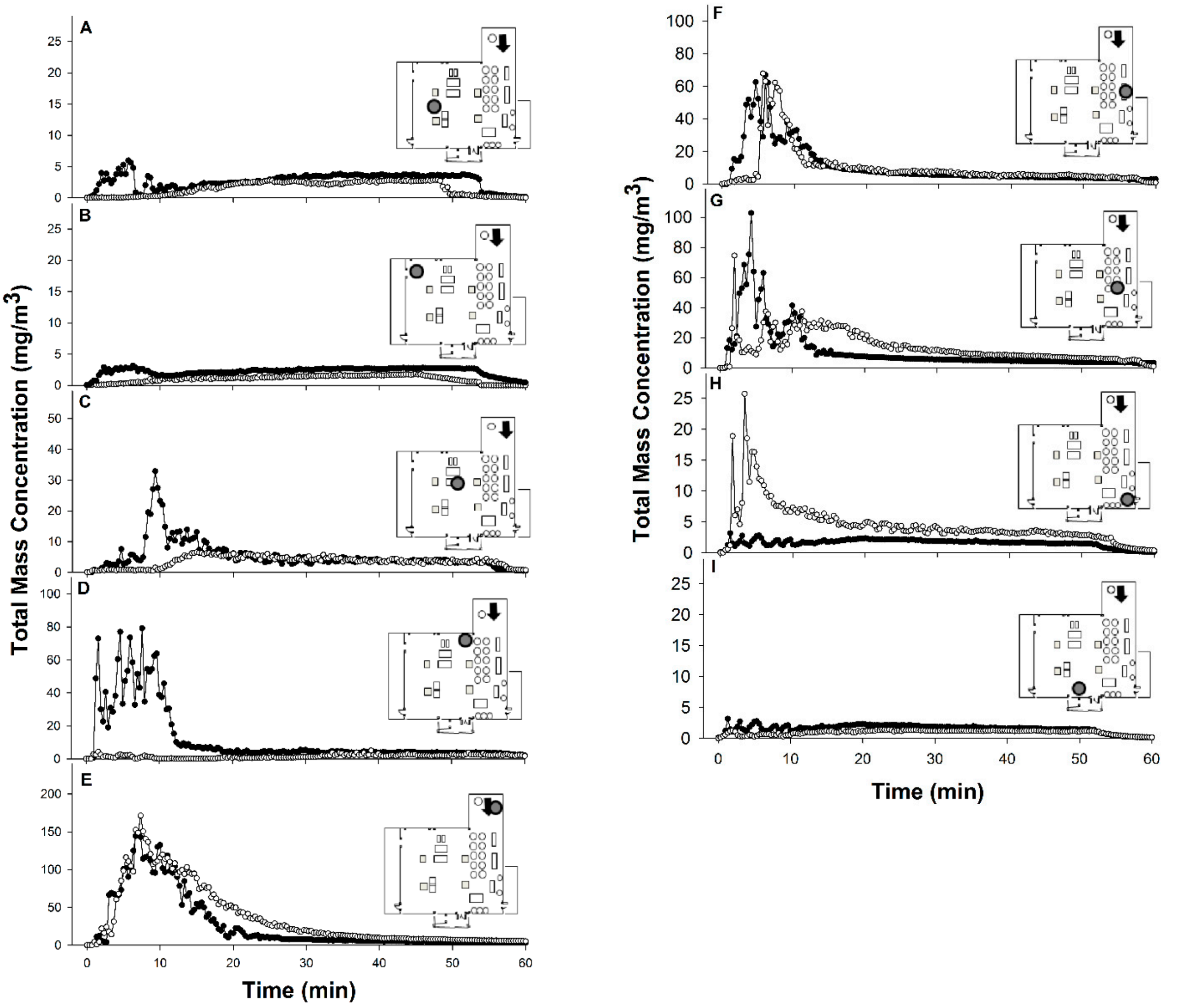

Figure S4b. Real time mass concentrations (mg/m^3) of **Pyrocide** aerosol particles detected by Aerodynamic Particle Sizers (APS) at nine locations after released from a portable hand-held fogger in a flour mill. The aerosol was released at the **north-west** location (shown with a solid arrowhead) and detected by APS in each of the nine locations (shown with a large gray circle) in each inserted floor plan (A-I). In each graph, the two lines represent the mass concentrations obtained for 20-s intervals over a 60 min-exposure to the aerosol in trial 1 (shown with solid circles) and trial 2 (shown with open circles).

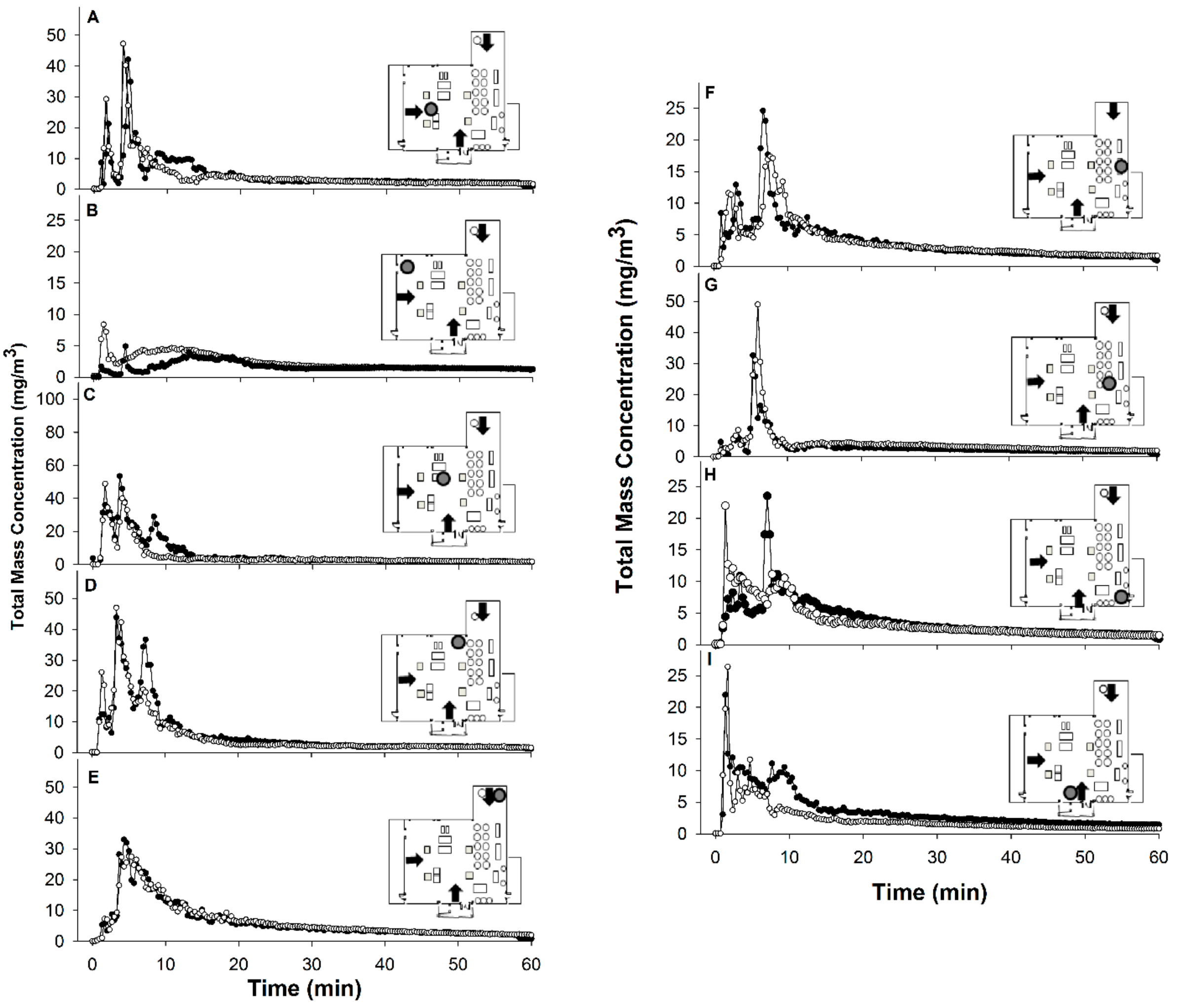

Figure S4c. Real time mass concentrations (mg/m^3) of **Turbocide** aerosol particles detected by Aerodynamic Particle Sizers (APS) at nine locations after released from a high pressure cylinder in a flour mill. The aerosol was released at **multiple** locations (shown with a solid arrowhead) and detected by APS in each of the nine locations (shown with a large gray circle) in each inserted floor plan (A-I). In each graph, the two lines represent the mass concentrations obtained for 20-s intervals over a 60 min-exposure to the aerosol in trial 1 (shown with solid circles) and trial 2 (shown with open circles).

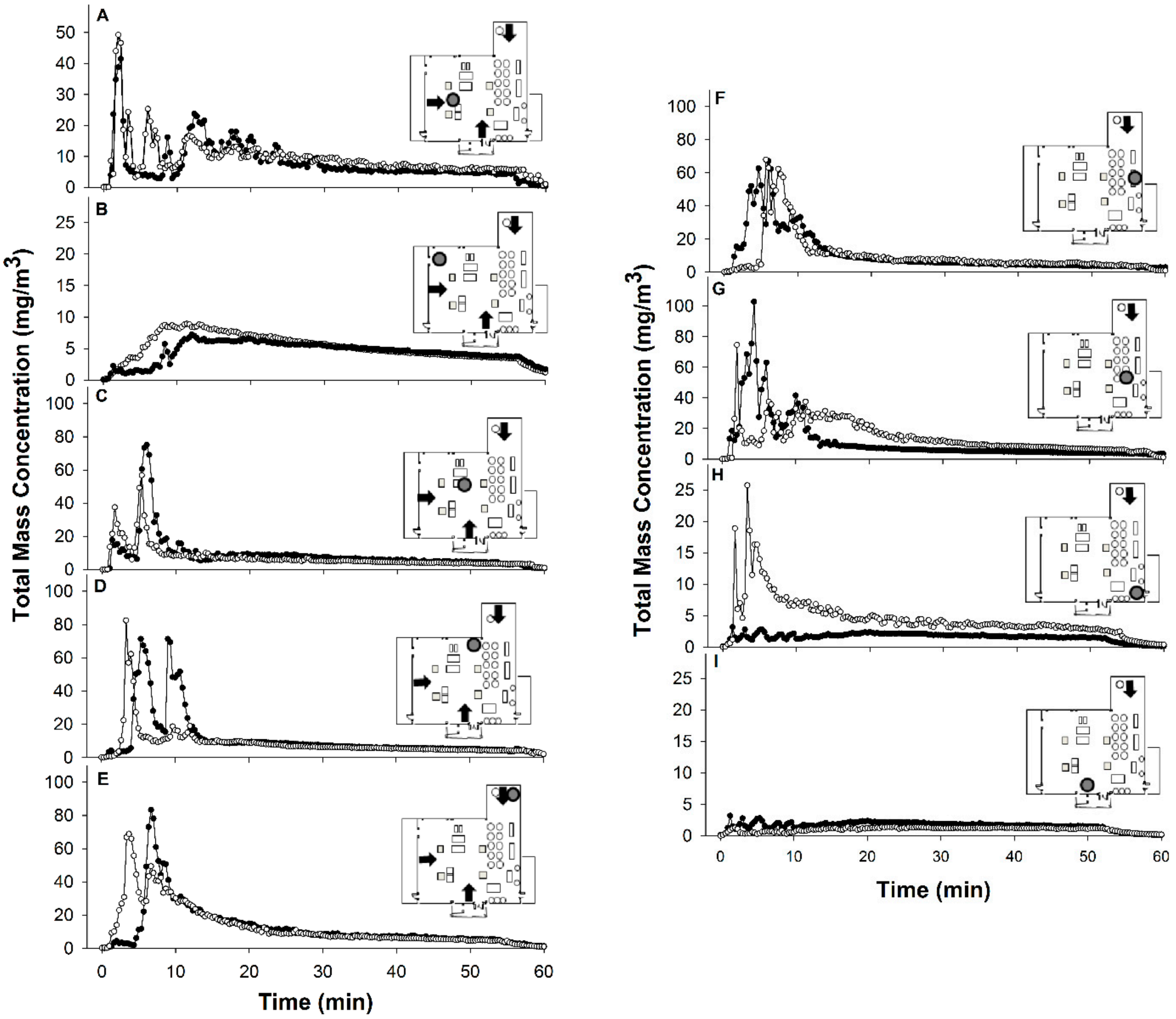

Figure S4d. Real time mass concentrations (mg/m^3) of **Pyrocide** aerosol particles detected by Aerodynamic Particle Sizers (APS) at nine locations after released from a hand-held portable fogger in a flour mill. The aerosol was released at **multiple** locations (shown with a solid arrowhead) and detected by APS in each of the nine locations (shown with a large gray circle) in each inserted floor plan (A-I). In each graph, the two lines represent the mass concentrations obtained for 20-s intervals over a 60 min-exposure to the aerosol in trial 1 (shown with solid circles) and trial 2 (shown with open circles).

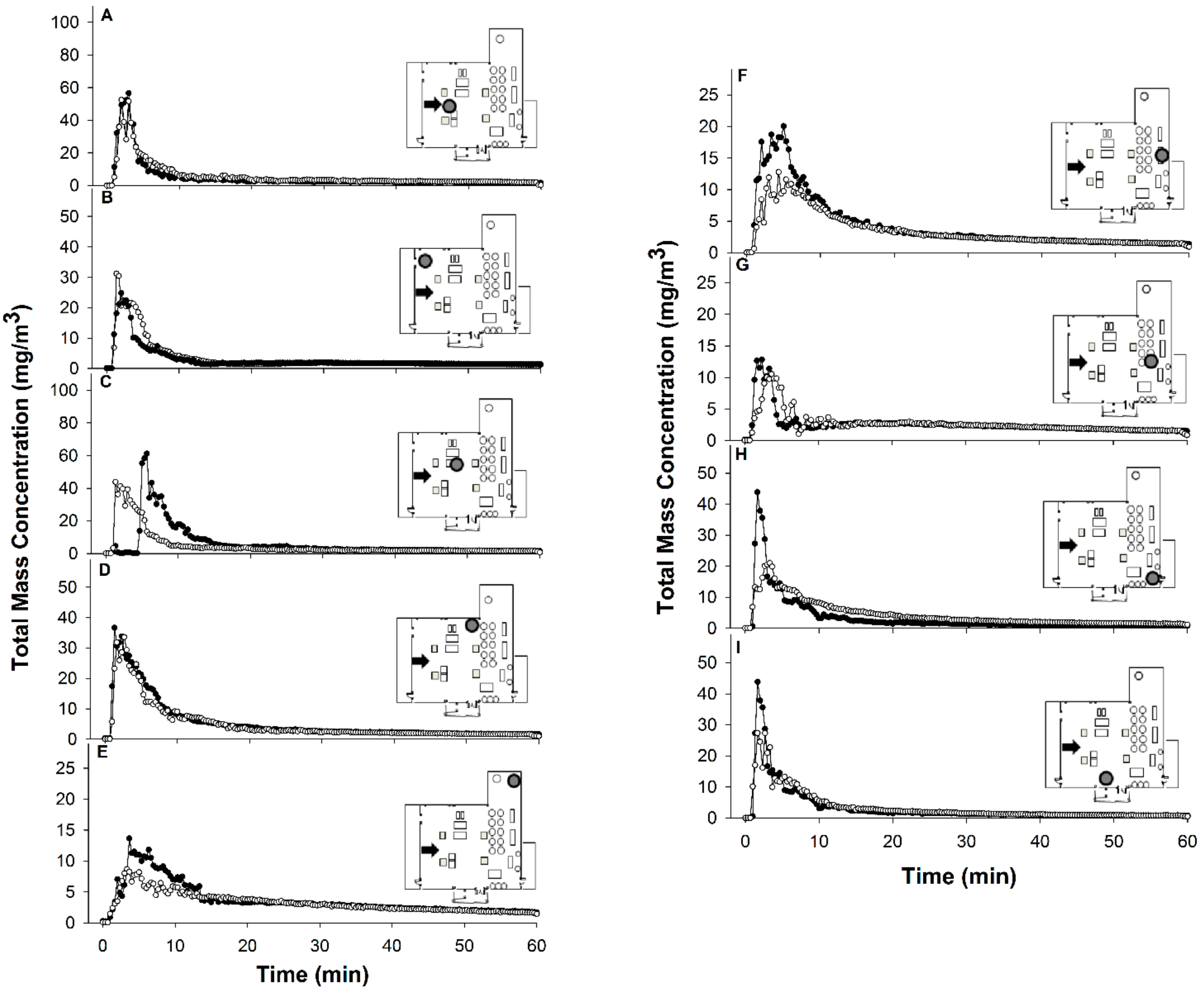

Figure S4e.Real time mass concentrations (mg/m^3) of **Turbocide** aerosol particles detected by Aerodynamic Particle Sizers (APS) at nine locations after released from a high pressure cylinder in a flour mill. The aerosol was released at **south** location (shown with a solid arrowhead) and detected by APS in each of the nine locations (shown with a large gray circle) in each inserted floor plan (A-I). In each graph, the two lines represent the mass concentrations obtained for 20-s intervals over a 60 min-exposure to the aerosol in trial 1 (shown with solid circles) and trial 2 (shown with open circles).

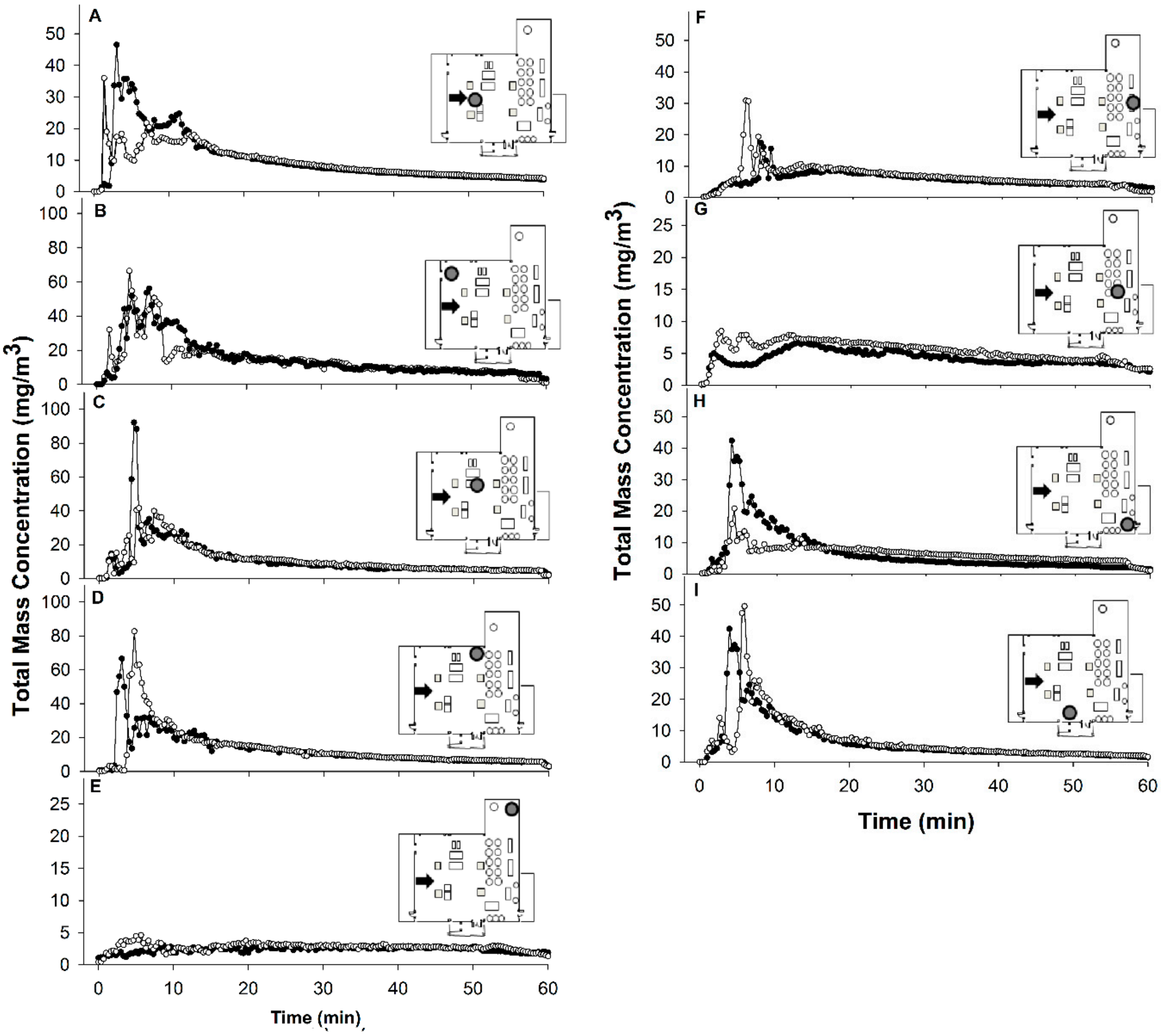

Figure S4f. Real time mass concentrations (mg/m^3) of **Pyrocide** aerosol particles detected by Aerodynamic Particle Sizers (APS) at nine locations after released from a hand-held portable fogger in a flour mill. The aerosol was released at **south** location (shown with a solid arrowhead) and detected by APS in each of the nine locations (shown with a large gray circle) in each inserted floor plan (A-I). In each graph, the two lines represent the mass concentrations obtained for 20-s intervals over a 60 min-exposure to the aerosol in trial 1 (shown with solid circles) and trial 2 (shown with open circles).

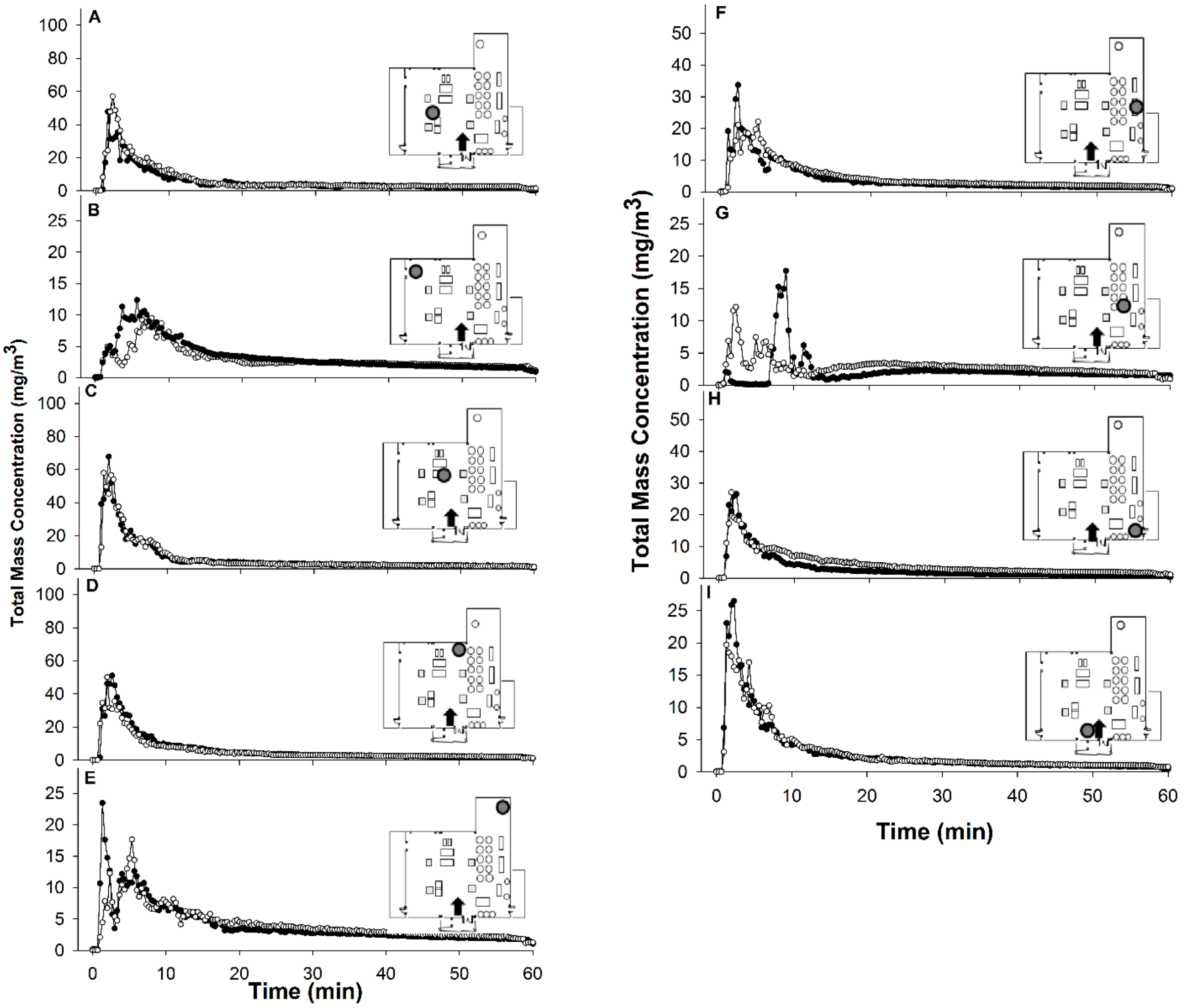

Figure S4g. Real time mass concentrations (mg/m^3) of **Turbocide** aerosol particles detected by Aerodynamic Particle Sizers (APS) at nine locations after released from a high pressure cylinder in a flour mill. The aerosol was released at **east** location (shown with a solid arrowhead) and detected by APS in each of the nine locations (shown with a large gray circle) in each inserted floor plan (A-I). In each graph, the two lines represent the mass concentrations obtained for 20-s intervals over a 60 min-exposure to the aerosol in trial 1 (shown with solid circles) and trial 2 (shown with open circles).

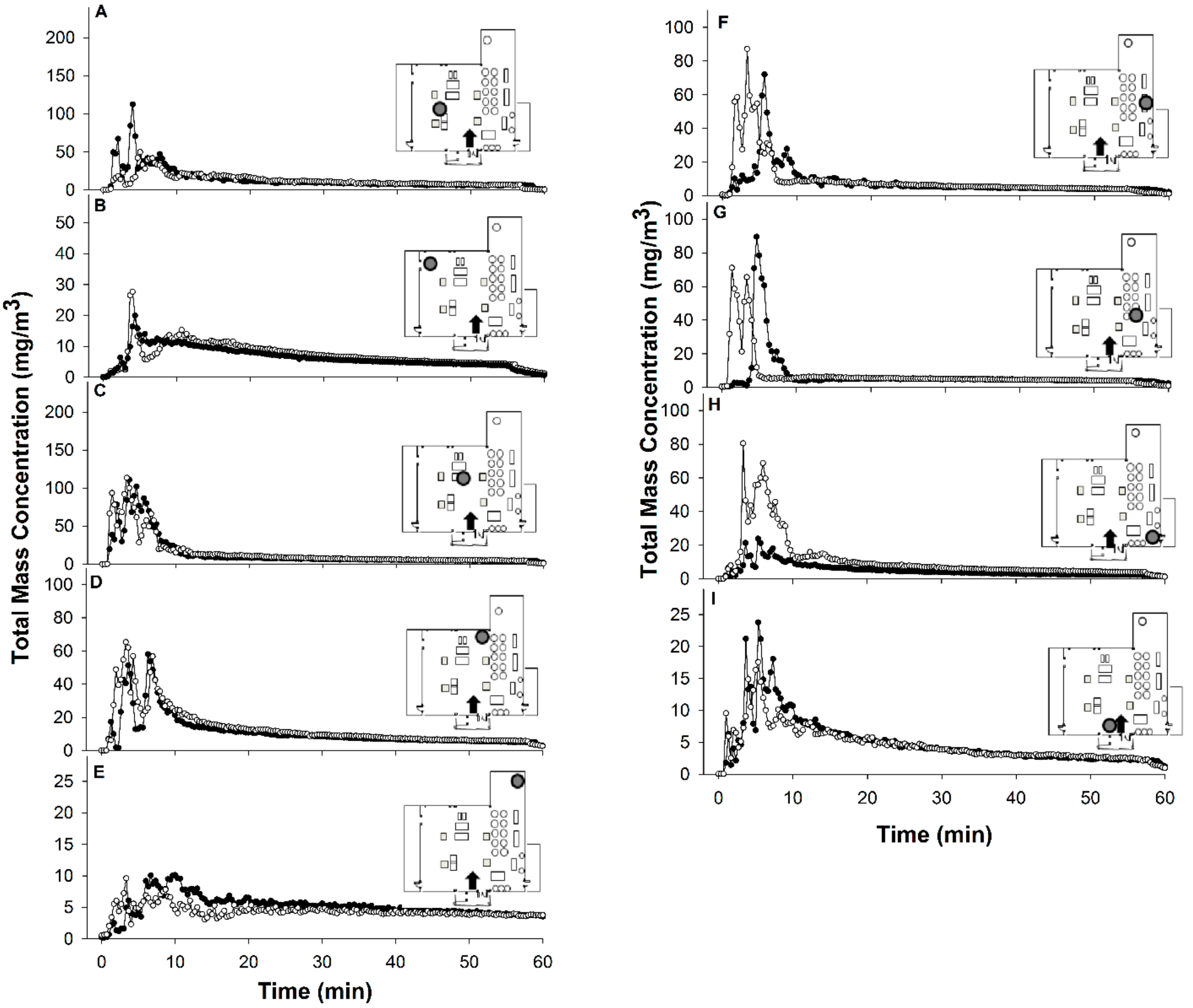

Figure S4h. Graphs showing real time mass concentrations of aerosol particles (mg/m3) obtained from nine Aerodynamic Particle Sizers (APS) at different locations in a flour mill exposed to **Pyrocide** aerosol released at **east** location (A-I). Each graph per APS unit with two lines represents mass concentrations obtained for 20 s intervals over a 60 minute-exposure to aerosol in trial 1 (black symbols) and trial 2 (white symbols). The inset in each graph is the floor plan of mill showing the location of APS unit (gray circle), and the location of Pyrocide aerosol released at east of the mill (black arrow) by using hand-held portable fogger.
